# Exploiting Phase Transitions in Catalysis: Adsorption of CO on doped VO_2_‐Polymorphs

**DOI:** 10.1002/cphc.202200131

**Published:** 2022-07-27

**Authors:** Berenike Stahl, Thomas Bredow

**Affiliations:** ^1^ Mulliken Center for Theoretical Chemistry Institute for Physical and Theoretical Chemistry University of Bonn Beringstr. 4 D-53115 Bonn Germany; ^2^ Mulliken Center for Theoretical Chemistry Institute for Physical and Theoretical Chemistry University of Bonn Beringstr. 4 D-53115 Bonn Germany

**Keywords:** physisorption, open-shell density functional calculations, vanadium oxide polymorphs, carbon monoxide, multireference methods

## Abstract

VO_2_ is well known for its low‐temperature metal‐insulator transition between two phases with tetragonal rutile and monoclinic structure. The adsorption of CO on the two polymorphs of Mo‐doped VO_2_ is calculated to investigate the effect of a substrate phase change on the adsorption energy. The system is investigated theoretically at density‐functional theory level using a hybrid functional with London dispersion correction. We establish a computational protocol applicable for the study of physisorption on open‐shell transition metal oxides. The main task is to control the spin state of open‐shell slab models used to model adsorption of closed‐shell molecules in order to obtain numerically stable adsorption energies and to reduce spin contamination within the broken‐symmetry unrestricted Kohn‐Sham approximation. Applying this procedure, it is possible to identify the most stable adsorption positions of CO on both phases of VO_2_. CO adsorbs vertically with the C atom on a surface V atom in the monoclinic phase with an adsorption energy of −56 kJ/mol. The same adsorption position has an adsorption energy of only −46 kJ/mol on the rutile phase. Similar differences were obtained with multireference methods using an embedded cluster model. This effect may inspire experimental strategies exploiting the rutile↔ monoclinic VO_2_ phase transition in catalytic processes where CO is formed as product or as an intermediate.

## Graphical abstract

**Adsorption properties of CO** on the surfaces of two VO_2_‐polymorphs are investigated using the M06 functional with D3‐correction. The spin state of the VO_2_‐surface is found to have a large influence on the adsorption properties, thereby unraveling a procedure to control the spin state of open‐shell slab models.

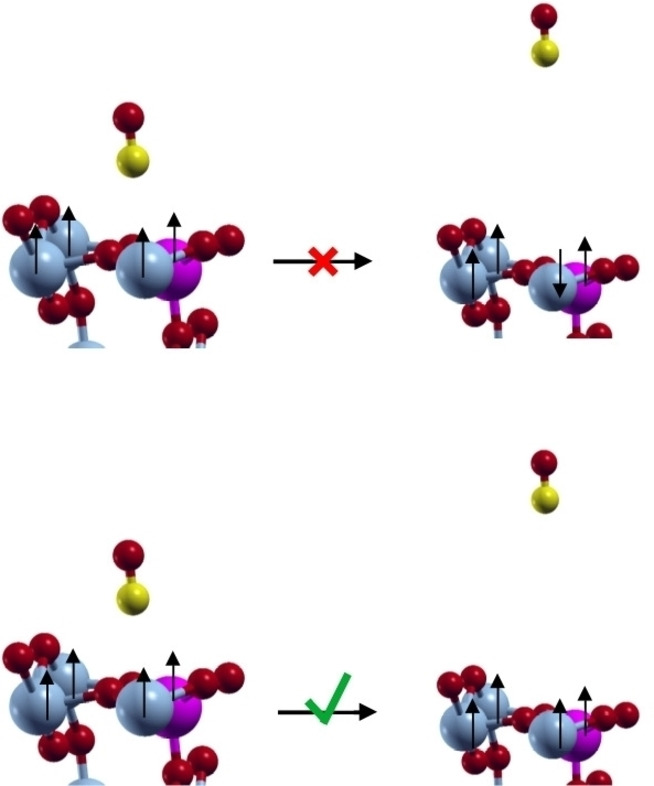

## Introduction

To meet the challenges of overall higher energy demand and the need for more sustainable resources faced by heterogeneous catalysis, many approaches to optimize industrial processes have been explored.[^1^, ^2^] For example, fluctuating reaction conditions in catalytic process are investigated to improve reaction conditions.^[2]^ In this way desorption of the product is facilitated, which would otherwise block the catalytically active sites. The present study investigates a phase transition of the substrate as an example of fluctuating reaction conditions. In order to improve the efficiency of a catalyst, the adsorption properties on the different phases of the catalyst should vary significantly. Then it would be possible to remove a product formed on the surface of one catalyst phase after transition to another phase which is less attractive.

In the present study we chose vanadium dioxide VO_2_ as substrate. Vanadium oxides have been investigated as catalyst materials because of their rich redox chemistry.^[3]^ VO_2_ in particular is catalytically active for the desulfurization of dibenzothiophene,^[4]^ the oxidative dehydrogenation of propane^[5]^ or the electrochemical reduction of trinitrotoluene.^[6]^

VO_2_ shows a metal‐insulator transition at 340 K between the tetragonal rutile and a monoclinic phase.[^7^, ^8^] The effect of this phase transition has been investigated for the catalytic oxidative desulfurization of dibenzothiophene.^[4]^ In the present study, we chose the adsorption of CO as a simple model system. CO is a product or an intermediate in many catalytic processes.[^9^, ^10^] The adsorption of CO on the most stable surfaces of both VO_2_ polymorphs is calculated in order to identify differences in the interaction strength which could be exploited in fluctuating reaction conditions for easier removal of the molecule.

Suitable density‐functional theory (DFT) methods for modeling the energetic, electronic and structural properties of the insulating monoclinic (M_1_) and the metallic rutile (R) phase of VO_2_ were identified in previous studies.[^11^, ^12^] A self‐consistent hybrid functional (sc‐PBE0) was found to provide the most accurate results for relative phase stability and electronic structure. Subsequently the surface properties of both VO_2_ polymorphs were investigated.^[13]^ A reconstruction of the R phase surfaces was found which has also been found experimentally.^[14]^ Doping with Mo stabilizes the R phase and preserves the structure of both phases also at the surface. The most stable surface models with the Mo dopant in the outer layers of the surface models were used in the present study.

The adsorption of molecules on surfaces containing open‐shell transition metals has to be calculated with spin‐polarized Kohn‐Sham DFT.^[15]^ This applies to the present system, since the VO_2_ M_1_ phase has an antiferromagnetic ground state according to Quantum‐Monte Carlo calculations,^[16]^ and the R phase is paramagnetic.[^16^, ^17^, ^18^] However, the mechanism of the metal‐insulator transition and the electronic structures of the phases are not fully understood by theory and experiment, as shown in a review article by Poguet.^[7]^ Electron‐electron interactions were found to play a decisive role in the transition mechanism. This underlines the importance of spin polarization in the theoretical treatment of this system. In this study, we show the importance of controlling the spin state for a numerically stable calculation of adsorption properties. Additionally, the effect of spin contamination due to the broken‐symmetry approximation is considered, which was discussed in previous studies.^[19]^

## Computational Details

All calculations were performed with the crystal‐orbital program CRYSTAL17 (version 1.0.2).^[20]^ The hybrid functional M06 with D3 dispersion correction[^21^, ^22^] was applied instead of the previously established sc‐PBE0 method.[^12^, ^13^] This was advantageous since M06 showed higher numerical stability in the adsorption calculations. Additionally, optimized D3 parameters are available for M06 which is not the case for sc‐PBE0. Inclusion of London dispersion was expected to be crucial for the calculation of adsorption energies. Results for the bulk phases of VO_2_ obtained with M06‐D3 and sc‐PBE0 are compared in the supplementary material Tables S1 and S2. M06‐D3 is only slightly less accurate than sc‐PBE0 for the calculation of lattice parameters, relative stability, and electronic structure.

Pob‐TZVP‐rev2 basis sets^[23]^ were used for V, O and C, and a pob‐TZVP basis set^[24]^ for Mo. The present basis sets are larger than those of our previous study.^[13]^ This proved to be necessary to ensure stable spin states and to reduce the basis set superposition error in the adsorption calculations. The integral truncation tolerances were set to the standard values of (10^−6^, 10^−6^, 10^−6^, 10^−6^, 10^−12^) to reduce computational effort. A Monkhorst‐Pack net with 12×12×1 k‐points was applied for the surface unit cells which was considered as converged. The optimized bulk lattice constants in Table S2 were used for the construction of surface models. To reduce computational cost only four‐layer slab models of Mo‐doped VO_2_ were used. The most stable surfaces R (110) and M_1_ (011) were investigated with one Mo atom each in the outermost layers of the slab. The R (110) surface is calculated with a 2×1 supercell which has the same size as the M_1_ (011) primitive unit cell. Both surface unit cells contain four metal and eight oxygen atoms per layer. The optimized bulk lattice constants were applied for the construction of surface models. Surface models were relaxed by optimization of all atomic positions under symmetry constraints with fixed lattice vectors, applying the quasi‐Newton algorithm implemented in CRYSTAL17^[20]^ unless indicated otherwise.

Ferromagnetic (FM), ferrimagnetic (FI) and antiferromagnetic (AFM) spin states were investigated for the surface models. Several methods were tested to set up the initial spin configuration of a system prior to the self‐consistent cycle. The keyword SPINLOCK constrains the number of electrons with up and down spin (*n*_*α*_–*n*_*β*_) until a predefined SCF convergence criterion (in the present calculations 10^−5^ a.u.) is reached. The spin states obtained in this way will be denoted as *fixed spin(x)* with *x=n*_*α*_–*n*_*β*_. An AFM state corresponds to *x*=0. The keyword FDOCCUPY allows to define individual initial occupation numbers for the *d* orbitals of V and Mo. For VO_2_, it was found that an initial occupation of the $d_{x^{2}-y^{2}}$ orbital leads to the most stable spin states. These calculations were denoted as *FDO*.

A counterpoise correction was applied to the calculated adsorption energies *E*_ads_ by replacing either the molecule or the slab atoms by ghost functions in single‐point energy calculations of the optimized geometry. The correction is in most cases relatively small, ≈10 kJ/mol, due to the use of BSSE‐corrected pob‐TZVP‐rev2 basis sets.

**Figure 1:**
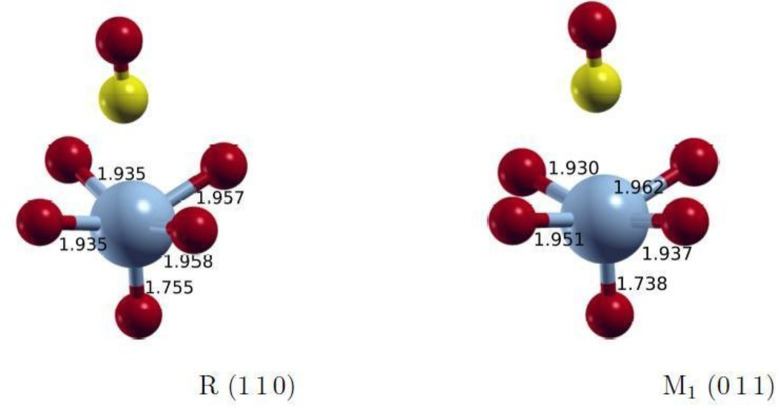
Structures of the clusters of the R (110) and M_1_ (011) surfaces; V: light blue, O: red, C: yellow.

FI and AFM states can only be set up using the broken‐symmetry approximation due to the use of single‐determinant wavefunctions in DFT. Since this is a crude approximation of the exact wavefunction, we additionally performed complete active space SCF calculations for comparison. CASSCF and NEVPT2 calculations were performed for finite embedded clusters shown in Figure 1 with ORCA version 4.2.1.[^25^, ^26^] Only the adsorption on V was considered in the comparison. The cluster calculations used def2‐TZVPP as well as def2‐QZVP^[27]^ basis sets and the auxiliary basis def2/JK.^[28]^ The clusters were embedded in a field of V‐atom ECPs^[29]^ and point charges. The point charge values were adjusted to the Mulliken charge of the central V atom, resulting in values +1.8 and −0.9. In the CASSCF calculations the five d‐orbitals of the V‐atom with one unpaired electron in the +IV oxidation state were considered to be in the active space. Only the lowest active CAS‐orbitals were considered in the NEVPT2 calculation.

## Results and Discussion

**Figure 2:**
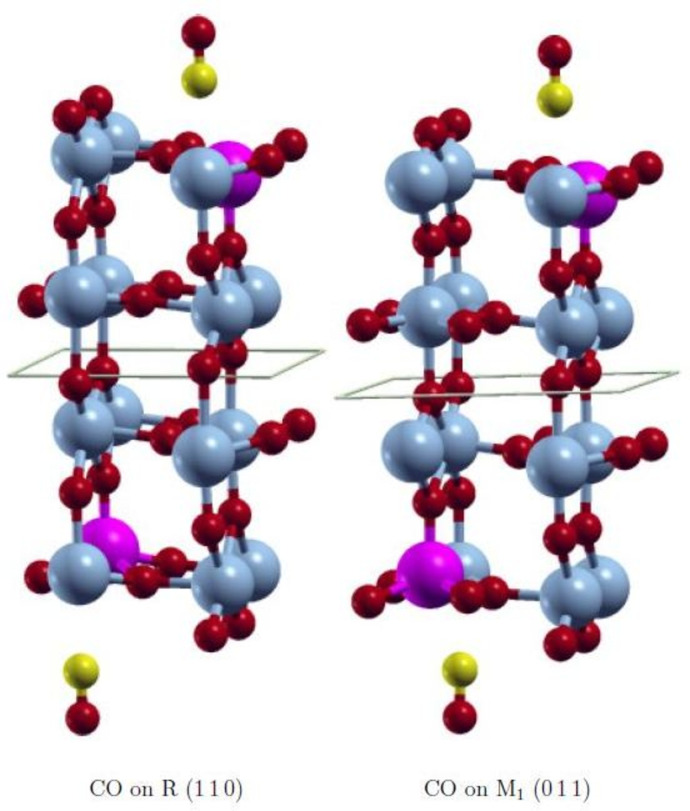
Optimized adsorption geometry of CO on the R (110) and M_1_ (011) surfaces. V: light blue, Mo: pink, O: red, C: yellow.

During preliminary calculations we found that the spin states of the slabs with and without adsorbate differed in an unpredictable way. Consequently, the calculated CO adsorption energies scattered significantly. Since the interaction of CO with V or Mo is weak to moderate, these changes in the spin state of the substrate are considered as artifacts of the self‐consistent field procedure. Additionally, adsorption energies obtained from closed‐shell calculations were found to vary significantly, even after small changes of the adsorbate structure (see Table S3). Therefore, we used various methods to control the spin configuration of the surface models and its effect on the adsorption energy (see below). The aim was to converge the slab to the same spin configuration with and without adsorbate, which should correspond to the magnetic ground state in both cases. The adsorption energies *E*_ads_ are calculated for CO adsorbed C‐down on a metal atom (see Figure 2). The resulting *E*_ads_ and the relative energy Δ*E*=*E* ^spin^−*E* ^groundstate^ to the spin state closest to the ground state are calculated and shown in Table 1.

**Table 1:** Calculated adsorption energy *E*_ads_ (kJ/mol) obtained for various spin configurations and their relative energy Δ*E* (kJ/mol) with respect to the most stable spin configuration for the surface with and without adsorbed CO; *FDO* denotes spin setting on the V and Mo atoms via the keyword FDOCCUPY; M06‐D3 results.

Spin					FDO w/o	FDO	FDO
setting	fixed spin(0)	fixed spin(8)	fixed spin(16)	fixed spin(18)	fixed spin	+fixed spin(0)	+fixed spin(18)
R							
*E*_ads_	88	−93	−19	−30	14	−52	−23
Δ*E* ^*Surf*^	75	310	160	22	29	132	0
Δ*E* ^*Surf+CO*^	281	116	171	16	134	93	0

M_1_							
*E*_ads_	−32	−65	−75	−27	−107	−70	−49
Δ*E* ^*Surf*^	169	244	239	0	265	56	0
Δ*E* ^*Surf+CO*^	190	175	177	34	165	27	0

We used various magnetic configurations of the Mo_2_V_14_O_32_ slabs (see Table 1) with a fixed geometry (see Figure 2). In the formal +IV oxidation state, each V atom has a *d* ^1^ configuration and Mo has a *d* ^2^ configuration. Therefore the highest sensible number of unpaired electrons is 18. We also defined an AFM spin configuration where the symmetry equivalent V and Mo atoms had opposite spin (fixed spin(0)). FI configurations with *x*=8 and *x*=16 were considered for comparison. The *x*=16 state is the most stable spin state for undoped VO_2_ surfaces. Some surface models were found to converge to a *x*=8 spin state where the three V‐atoms and the Mo‐atom in the topmost layers showed an antiferromagnetic configuration while the inner layers showed ferromagnetic ordering. Therefore, the *x*=8 state was also chosen. Manual spin setting for individual metal *d* orbitals (using the CRYSTAL keyword FDOCCUPY, denoted as FDO) was tested without spin locking, and with fixed spin(0) and fixed spin(18). The FM fixed spin(18) wavefunction with FDO was found to be the most stable spin configuration in both phases with and without the adsorbate. All other attempts resulted in SCF solutions that were up to 310 kJ/mol higher in energy. The stability of the spin states has a large influence on the adsorption energy. An unstable spin state in the surface without adsorbate leads to a large *E*_ads_. This can be seen in the rutile phase for the fixed spin(8) state. The surface is 310 kJ/mol less stable than the fixed spin(18) state with FDO, while the model with adsorbate was 116 kJ/mol less stable. After counterpoise correction this leads to a strongly negative *E*_ads_ of −93 kJ/mol. This can also be observed in the M_1_ phase model calculated with FDO without fixed spin. The surface in this state is 265 kJ/mol less stable than the fixed spin(18) state with FDO. This leads to *E*_ads_ of −107 kJ/mol. On the other hand, an unstable spin state of the surface with adsorbate leads to a small or positive adsorption energy. This can be observed in the AFM fixed spin(0) and FDO (w/o fixed spin) state of the R phase. These states yield *E*_ads_ of +88 and +14 kJ/mol, respectively. These results show the importance of finding the most stable spin state when calculating adsorption energies of open shell systems.

A previous study of Biz et al.^[15]^ showed the importance of spin‐polarization in calculations of surface adsorption due to exchange interaction. Our results show, that furthermore the magnetic states of the substrate and the substrate‐adsorbate system must be carefully controlled in order to obtain physically meaningful results.

Due to the use of the broken‐symmetry approximation, most wavefunctions suffer from spin contamination. They are not eigenfunctions of the total spin operator, but contain contributions from other spin states. An energy correction with respect to spin contamination, as discussed by Tada et al.,^[19]^ is not available in the CRYSTAL17 program. However, the spin contamination can be calculated as the difference of the actual S^2^‐value yielded by the calculation and the ideal S^2^‐value of the system. The results are shown in Table 2. The spin contamination decreases with increased ferromagnetic ordering of the spins. The AFM states show large spin contamination of about 8.5–9 in both phases. This is a result of the single‐determinant ansatz to model multiconfiguration electronic states. Nevertheless, the addition of the keyword FDOCCUPY results in a decrease of the spin contamination to about 8.2. The spin contamination of the FDO (w/o fixed spin) results depend on the spin state the calculation converges to. The ferromagnetic state with increased electron number in combination with FDO yields a reduced spin contamination of ≈0.5 in all models.

**Table 2:** Spin contamination of the 4‐layer M_1_ (011) and R (110) slab without (*Surf*) and with adsorbed CO (*Surf+CO*); M06‐D3 results.

Spin					FDO w/o	FDO	FDO
setting	fixed spin(0)	fixed spin(8)	fixed spin(16)	fixed spin(18)	fixed spin	+fixed spin(0)	+fixed spin(18)
R							
Surf	9.39	6.62	2.18	0.94	0.85	8.21	0.60
Surf+CO	8.57	5.71	1.85	0.93	2.66	8.21	0.43

M_1_							
Surf	9.12	4.31	1.56	0.50	0.56	8.25	0.50
Surf+CO	8.95	5.61	1.76	0.49	2.47	8.26	0.48

**Figure 3:**
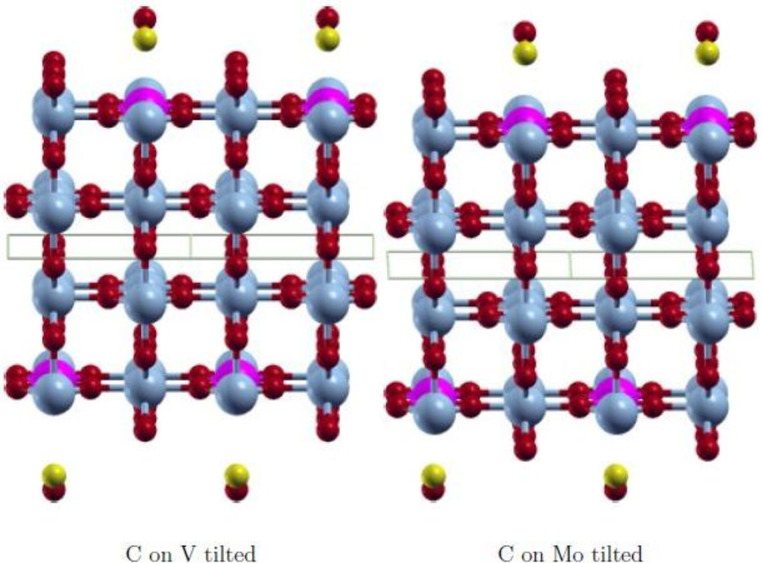
Initial *tilted* adsorption structure with CO C‐down and oriented along [10]. V: light blue, Mo: pink, O: red, C: yellow.

**Figure 4:**
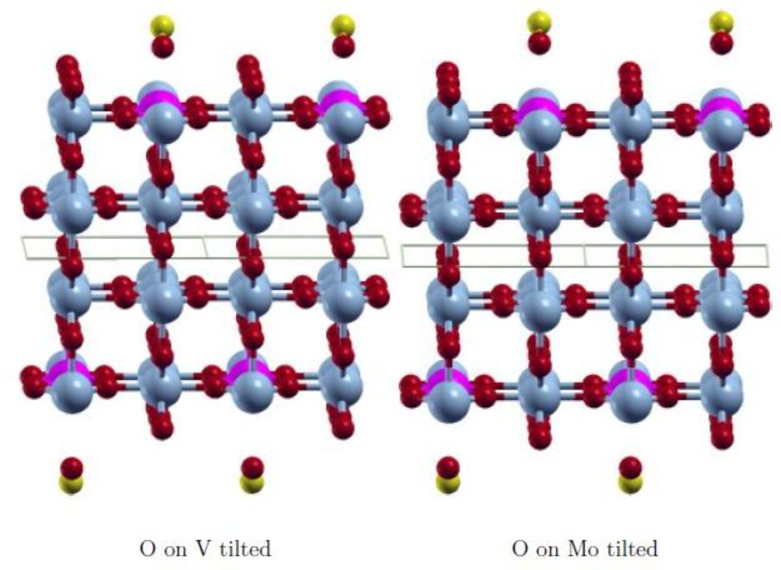
Initial *tilted* adsorption structure with CO O‐down and oriented along [10]. V: light blue, Mo: pink, O: red, C: yellow.

**Figure 5:**
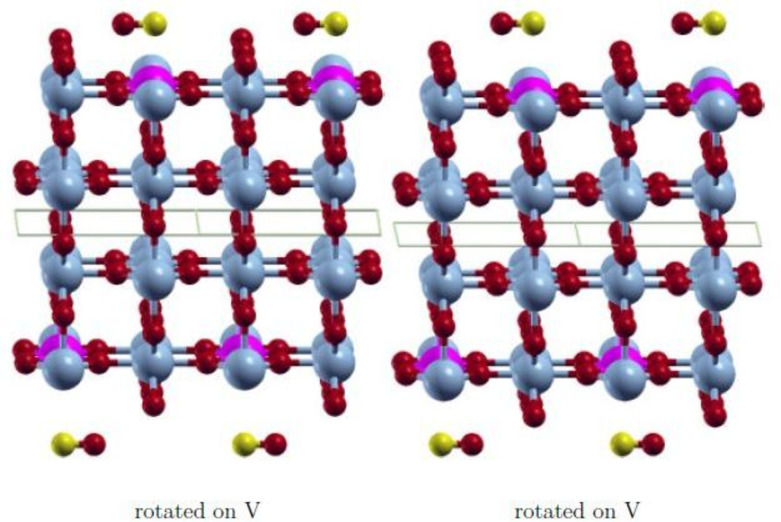
Initial *rotated* adsorption structure with CO oriented along [10]. V: light blue, Mo: pink, O: red, C: yellow.

**Figure 6:**
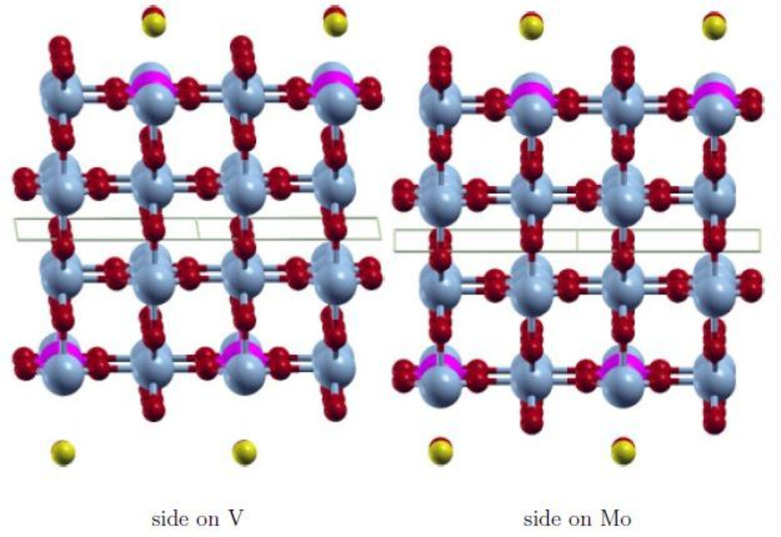
Initial *side‐on* adsorption structure with CO O‐down and oriented along [10]. V: light blue, Mo: pink, O: red, C: yellow.

The wavefunction obtained with FDO+fixed spin(18) is in all cases the most stable state and has the smallest spin contamination. Therefore, this approach was used to investigate various positions of the adsorbate CO to find the most stable adsorption state. The initial positions on the surface V‐ and Mo‐atom are shown in Figures 3, 4, 5, 6. They are equivalent on both polymorph surfaces. Similar to a previous study of CO and CO_2_ adsorption on doped TiO_2_ rutile,^[30]^ we tested vertical adsorption C‐down on V and Mo, side‐on adsorption (*side*), a tilted structure (*tilted*) and a rotated structure (*rotated*). The resulting adsorption energies as well as the optimized distance between the adsorbate and the surface d_*ads*_ are shown in Table 3. In Figures 3, 4, 5, 6 the initial adsorption structures are shown for *side*, *tilted* and *rotated* positions. As in our previous study,^[13]^ the slabs are symmetric in order to avoid artificial dipole moments. Therefore two CO molecules are adsorbed on the top and bottom layers.

**Table 3:** Adsorption energies *E*_ads_ of various CO adsorption positions (kJ/mol), and distance d_*ads*_ (Å) between the surface and adsorbate; M06‐D3 results with counterpoise correction.

Adsorbate position	R		M_1_	
	*E*_ads_	d_*ads*_	*E*_ads_	d_*ads*_
vertical C on V	−23	2.44	−49	2.34
vertical C on Mo	−60	2.70	−43	2.59
vertical O on V	−8	2.64	−25	2.49
vertical O on Mo	0	2.81	−9	2.75

**Figure 7:**
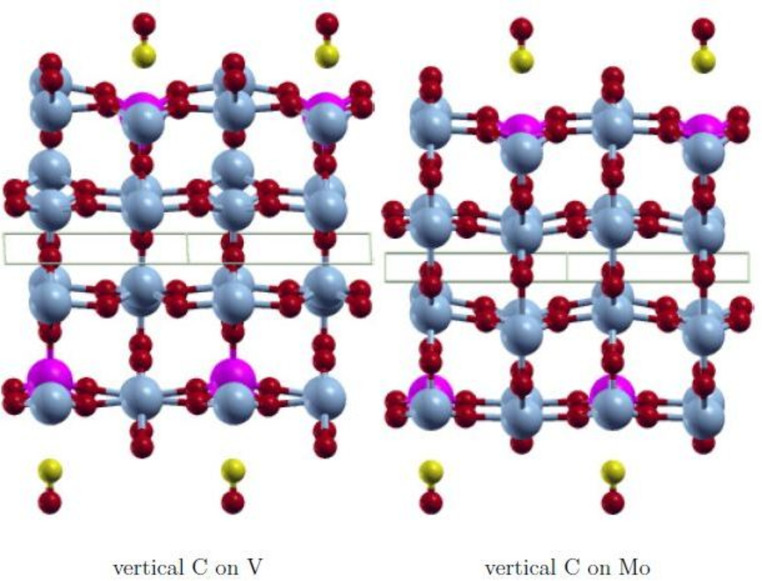
Optimized CO adsorption geometries on the R (110) surface. V: light blue, Mo: pink, O: red, C: yellow.

All initial CO positions C on V/Mo *tilted*, *side* and *rotated* converged to the same, essentially vertical, adsorption structure shown in Figure 7. The O atom of CO only weakly interacts with the metal atoms of the surfaces, Table 3. Generally, the C−M distance d_*ads*_ is larger on Mo than on V. d_*ads*_ is about 2.3–2.5 Å on V and about 2.6–2.7 Å on Mo. On the R surface, the adsorption on Mo is the most stable adsorption position with *E*_ads_=−60 kJ/mol. The adsorption on V and Mo on the M_1_ surface yield similar adsorption energies of −49 and −43 kJ/mol, respectively. Projected densities of states (pDOS) were calculated for all of the calculated adsorption structures (see Figure S1–S10 in the supplementary material). Only the *α* (spin‐up) frontier orbitals are shown which are occupied by the unpaired electrons of V and Mo. The calculated pDOS of the surfaces are similar to the pDOS obtained with sc‐PBE0 in a previous study.^[13]^ The fundamental band gap calculated with M06‐D3 is larger compared to the sc‐PBE0 results, due to the higher amount of Fock‐exchange (27 % compared to 12.7 %). CO adsorption leads to small changes of the surface pDOS, as expected for physisorption. The Fermi energy is slightly shifted to higher values. Small contributions of the C‐orbitals are found in the frontier orbitals. The R phase shows a slight localization of the V‐d‐orbitals near the Fermi level for adsorption of CO on the V‐atom. This is not observed in the M_1_ phase, where the V‐d‐orbitals near the Fermi level are already localized. The increase of the band width of the metal d‐orbitals due to CO adsorption is more pronounced when the surface is calculated using fixed spin(18) w/o FDO (see Figure S3). This effect is probably responsible for the observed scattering of the calculated adsorption energies.

Up to this point, the adsorption energies were calculated in the usual way by subtracting the energies of the separated systems from the energy of the adsorbate system (Eq. 1.

(1) $$E{}_{\mathrm{ads}}=E\left(\mathrm{slab}\;:\;\mathrm{CO}\right)-E\left(\mathrm{slab}\right)-E\left(CO\right)$$

**Figure 8:**
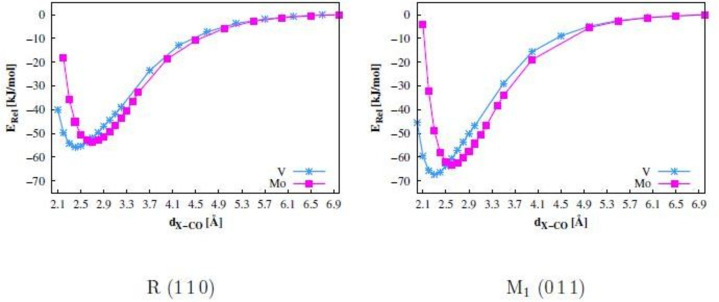
Potential energy curves for vertical C‐down CO adsorption on the V‐ and Mo‐atom on the R (110) and M_1_ (011) surfaces; M06‐D3 results without counterpoise correction.

The most stable C‐down vertical adsorption position was calculated with a different spin control ansatz to obtain more accurate adsorption energies, because the atomic spin densities still differ between the bare and CO‐covered slab, especially in the R phase (see Table 5). We started from the minimum adsorbate structure shown in Figure 7 and gradually increased the C−M distance, keeping the vertical CO orientation fixed. For every point the C−O distance was relaxed, but the atoms of the slab were fixed at the initial minimum positions. Test calculations showed that relaxation of the surface atoms led to changes in the spin configuration, in particular for the R phase. Applying these constraints it was possible to stabilize the atomic spin distribution over the full distance range between the minimum structure and the separated systems with C−M=7 Å. The adsorption energies in this second ansatz are defined as difference between the energy plateau at C−M=7 Å, and the minimum energy. This is much more consistent in terms of spin configuration conservation than the usual reference of separated slab and CO according to Eq. 1. The resulting potential curves (not corrected for BSSE) are shown in Figure 8. The counterpoise correction was calculated only for the minimum structures of the potential energy curves (see Table 4). It can be safely assumed that the separated systems have no BSSE. For comparison, the previous adsorption energies of Table 3 (now denoted as $E_{\mathrm{ads}}^{\mathrm{prev}}$ ) are also shown.

**Table 4:** Adsorption energies *E*_ads_ of CO on V and Mo atoms of the R (110) and M_1_ (011) surfaces in kJ/mol obtained as difference between minimum and plateau energies in Figure 8 and after counterpoise correction of the minimum structures; M06‐D3 results.

Phase	*E*_ads_		$E_{\mathrm{ads}}^{\mathrm{prev}}$	
	on V	on Mo	on V	on Mo
M_1_	−56	−52	−49	−43
R	−46	−43	−23	−60

**Table 5:** Mulliken spin population of the V and Mo atoms of the 4‐layer R (110) and M_1_ (011) slab without (*Surf*) and with adsorbed CO (*Surf+CO*); M06‐D3 results obtained with FDO+fixed spin(18). The atom, on which CO is adsorbed, has been highlighted.

Rut	1st layer				2nd layer			
	V	Mo	V	V	V	V	V	V
Surf	**1.38**	1.01	1.23	1.50	1.43	1.33	2.11	1.43
Surf+CO	**2.16**	0.99	0.24	1.27	1.34	1.36	1.68	1.96

M_1_	1st layer				2nd layer			
	V	Mo	V	V	V	V	V	V
Surf	**2.16**	1.31	1.26	0.99	1.38	1.36	1.37	1.40
Surf+CO	**2.22**	1.30	1.26	0.97	1.37	1.35	1.36	1.40

The CO adsorption energies obtained from the potential curves of the M_1_ surface are similar to the previous results. After counterpoise correction similar adsorption energies of −56 and −52 kJ/mol are obtained above V and Mo, respectively. The adsorption energies are slightly lower than the previous results of −49 kJ/mol on V and −43 kJ/mol on Mo. For the R phase, an adsorption energy of −46 kJ/mol is calculated for the adsorption on the V‐atom. This result is significantly lower than the −23 kJ/mol yielded previously. The adsorption energy on the Mo‐atom changed from −60 kJ/mol to −43 kJ/mol. These differences can be explained by the spin distribution changes in the bare and CO‐covered slab when using Eq. 1. The much higher consistency of the spin configurations obtained with the second ansatz is seen in Tables 6 and 7. Changes of the atomic magnetizations between minimum distance and quasi‐separated systems are negligible.

**Table 6:** Mulliken spin population of the V and Mo atoms of the 4‐layer R (110) slab with adsorbed CO at various distances; M06‐D3 results obtained with the second ansatz. The atom on which CO is adsorbed has been highlighted.

on V	1st layer				2nd layer			
	V	Mo	V	V	V	V	V	V
2.4 Å	**2.16**	0.99	0.24	1.27	1.34	1.36	1.68	1.96
3.5 Å	**2.15**	0.99	0.23	1.26	1.34	1.36	1.68	1.95
7.0 Å	**2.15**	0.99	0.23	1.26	1.34	1.36	1.68	1.95

on Mo	1st layer				2nd layer			
	V	Mo	V	V	V	V	V	V
2.7 Å	1.39	**0.99**	1.28	1.37	1.34	1.41	1.40	2.13
3.5 Å	1.39	**0.99**	1.28	1.38	1.34	1.41	1.40	2.13
7.0 Å	1.39	**0.99**	1.28	1.38	1.34	1.41	1.40	2.13

**Table 7:** Mulliken spin population of the V and Mo atoms of the 4‐layer M_1_ (011) slab with adsorbed CO at various distances; M06‐D3 results. The atom on which CO is adsorbed has been highlighted.

on V	1st layer				2nd layer			
	V	V	V	Mo	V	V	V	V
2.3 Å	**2.22**	1.30	1.26	0.97	1.37	1.34	1.35	1.40
3.5 Å	**2.21**	1.30	1.26	0.98	1.37	1.34	1.35	1.40
7.0 Å	**2.21**	1.30	1.26	0.98	1.37	1.34	1.35	1.40

on Mo	1st layer				2nd layer			
	V	V	V	Mo	V	V	V	V
2.6 Å	2.14	0.21	2.13	**1.00**	1.41	1.37	1.35	1.37
3.5 Å	2.14	0.20	2.13	**1.00**	1.41	1.37	1.35	1.37
7.0 Å	2.14	0.20	2.13	**1.00**	1.41	1.37	1.35	1.37

With the second ansatz, we found a difference of ≈10 kJ/mol between the adsorption energies on the R and M_1_ phases (see Table 4). The equilibrium CO partial pressure on the surfaces was estimated to investigate the possibility of exploiting the phase transition for CO removal. This was done using the equation based on the Gibbs–Helmholtz:^[31]^

$$p/p{}_{0}=e^{E_{\mathrm{ads}}/RT}$$

where *G*_ads_ was approximated by *E*_ads_. The CO partial pressure *p* was calculated at various temperatures for the adsorption on V. In real systems the influence of the dopant Mo, which is present only in small amounts, is assumed to be negligible. The estimated partial pressures in bar are shown in Table 8. At 273.15 K, the partial pressure needed to desorb CO from the R surface is two orders of magnitude higher than on the M_1_ surface. One order of magnitude difference between the phases is still present at 473.15 K. This is an indication that CO removal after M_1_→R phase transition is possible. A reaction with CO as the product may take place on the M_1_ surface. Afterwards, the temperature can be raised so that the phase transition to the R phase takes place, where CO could more easily desorb. It has to be noted, that these properties could change with increased CO coverage. Further research on this issue is required.

**Table 8:** Estimated CO partial pressure in bar on M_1_ and R surfaces at a given temperature T in K. Adsorption structures with CO C‐down on V were considered.

Phase	T		
T	273.15	373.15	473.15
M_1_	1.95×10^−11^	1.45×10^−8^	6.57×10^−7^
R	1.59×10^−9^	3.64×10^−7^	8.35×10^−6^

As mentioned above, the accuracy of slab calculations is limited due to the use of a single‐determinant wavefunction. With the proposed approach of applying restrictions to the slab geometry and spin configuration with FDO and SPINLOCK in sequential calculations of a potential curve, the corresponding errors cancel out to a large extent. But still the reliability of this approach needs to be assessed by comparison with higher‐quality methods. Therefore, the CO adsorption was calculated with embedded cluster models at NEVPT2 level. The resulting adsorption energies, again obtained from potential curve scans, are shown in Table 9.

**Table 9:** Comparison of adsorption energies *E*_ads_ (kJ/mol) calculated with NEVPT2/def2‐TZVPP, NEVPT2/def2‐QZVP and M06‐D3.

	R	M_1_
	*E*_ads_	
def2‐TZVPP	−53	−37
def2‐QZVP	−76	−89
M06‐D3 (ansatz 2)	−46	−56

The NEVPT2 results show a strong dependence on the basis set quality. This is mainly due to the basis set incompleteness, the NEVPT2 results therefore include a BSSE, which could not be corrected. The results with the larger def2‐QZVP basis set where BSSE is usually small, are more considered as more reliable. The relative stability of CO on R and M_1_ obtained with NEVPT2/def2‐QZVP and M06‐D3 is similar, although the absolute values are larger by ≈30 kJ/mol with the former.

The NEVPT2 results give insight into the orbitals that participate in the V–CO interaction. The Mulliken population analysis showed that mainly the V $d_{x^{2}-y^{2}}$ orbital is occupied in both phases. This is in accordance with the results of the surface models with M06‐D3. Overall, the comparison with NEVPT2/def2‐QZVP results confirms the validity of our second ansatz.

## Conclusions

We investigated the adsorption of CO on the most stable surfaces of the R and M_1_ phases of VO_2_. It was found that controlling the atomic magnetizations in order to identify the most stable spin configuration is crucial to obtain numerically stable and therefore reliable adsorption energies, within the limitations of DFT. For both surfaces a ferromagnetic state was found to be the most stable spin state, which also has the smallest spin contamination within the single‐determinant approximation. Control of the initial *d* orbital occupation was found to further reduce spin contamination. Restricted‐geometry CO‐surface potential curves were calculated for the most stable spin state. By fixing the slab geometry, its spin state could be stabilized which led to numerically stable adsorption energies, that were semi‐quantitatively reproduced with a multireference approach. We suggest this procedure for the study of physisorption on open‐shell systems, which is particularly suited for atom‐centered basis sets. The most stable CO adsorption position on both phases was vertical C‐down on the transition metals. An adsorption energy of −46 kJ/mol on the R phase and −56 kJ/mol on the M_1_ phase were obtained on V. A similar difference is calculated with NEVPT2 and quadruple‐zeta basis sets. Thus it may be possible to exploit the R↔M_1_ phase transition in a catalytic reaction where CO is a product or an intermediate of a reaction on M_1_ and desorbs from R after moderate heating.

## Conflict of interest

The authors declare no conflict of interest.

1

## Supporting information

As a service to our authors and readers, this journal provides supporting information supplied by the authors. Such materials are peer reviewed and may be re‐organized for online delivery, but are not copy‐edited or typeset. Technical support issues arising from supporting information (other than missing files) should be addressed to the authors.

Supporting Information

## Data Availability

The data that support the findings of this study are available in the supplementary material of this article.
