# Supplementary material for: Exploiting Phase Transitions in Catalysis: Adsorption of CO on doped VO2‐Polymorphs
Source: Chemphyschem. 2022 Jul 27;23(20):e202200131. doi: 10.1002/cphc.202200131 (PMC9796616; doi:10.1002/cphc.202200131)
Supplement: Supplementary file 1 — Supporting Information [file CPHC-23-0-s001.pdf]

ChemPhysChem

Supporting Information

Exploiting Phase Transitions in Catalysis: Adsorption of CO on doped VO_2-Polymorphs

Berenike Stahl* and Thomas Bredow*

1 Supplementary Information

Table S1: Calculated band gaps in eV, and V-V distances in Å obtained with sc-PBE0, $\alpha = 12.7\%(\mathrm{M}_1), 0.0032\%$ (R); and M06-D3 in a ferromagnetic state; the relative phase stability ΔE_{M_1-R} (eV) is calculated with $\alpha = 12.7\%$ for both phases.

	(M_1)		(R)		
Spin state	band gap	V-V distances	band gap	V-V distance	ΔE_{M_1-R}
M06-D3	1.80	2.88, 3.14	0.01	2.86	-0.17
sc-PBE0[a]	1.06	2.87, 3.15	0.00	2.84	-0.11
Exp.	0.60[b]	2.65, 3.12[c]	0.0[b]	2.87[e]	-0.044[c], -0.008[d]

[a]: Ref.[1] [b]: Ref. [2], [c]: Ref. [3], [d]: Ref. [4], [e]: Ref. [5]

Table S2: Results for lattice parameters of the M_1 and R phases of VO_2 in Å and ° as obtained with sc-PBE0, $\alpha = 12.7\,\%$ (M_1), $\alpha = 0.0032\,\%$ (R) and M06-D3 in an ferromagnetic state. The mean relative deviation (MRD) and the mean absolute relative deviation (MARD) to the experimental parameters are given in %. For the fractional coordinates, mean deviations (MD) and mean absolute deviations (MAD) are given.

	M06-D3		sc-PBE0[a]		Exp.	
	M_1	R	M_1	R	M_1^b	R^c
a	6.011	5.716	6.009	5.679	5.753	5.702
b	4.447	4.527	4.464	4.569	4.526	4.552
c	5.345	5.353	5.346	5.379	5.383	5.371
β	124.1	122.3	123.9	121.9	122.6	122.1
u_{V}	0.2465	0.2500	0.2525	0.2500	0.2403	0.2500
v_{V}	0.0140	0.0000	0.0161	0.0000	0.0180	0.0000
w_{V}	0.0151	0.0000	0.0185	0.0000	0.0318	0.0000
u_{O1}	0.1029	0.0992	0.1025	0.1002	0.1060	0.0975
v_{O1}	0.2003	0.1983	0.2010	0.2000	0.2100	0.1950
w_{O1}	0.1972	0.1984	0.1947	0.2004	0.2030	0.1950
u_{O2}	0.4021	0.4008	0.4031	0.3998	0.4160	0.4025
v_{O2}	0.7019	0.6983	0.6996	0.7000	0.7350	0.6950
w_{O2}	0.2923	0.3016	0.2918	0.2996	0.3160	0.3050
$\mathrm{MRD}_{abc\beta}$	0.8	-0.1	0.9	0.0		
$\mathrm{MARD}_{abc\beta}$	2.0	0.3	1.9	0.3		
MD_{uvw}	−0.0146	0.0007	−0.0107	0.0011		
MAD_{uvw}	0.0160	0.0017	0.0134	0.0029		

[a]: Ref.[1] [b]: Ref.[6], [c]: Ref.[7]

Table S3: Adsorption energies E_{ads} of various CO adsorption positions (kJ/mol), and distance d_{ads} (Å) between the surface and adsorbate; closed shell M06-D3 results with counterpoise correction.

Adsorbate position	R		M_1	
	E_{ads}	d_{ads}	E_{ads}	d_{ads}
vertical C on V	−51	2.53	−33	2.34
vertical C on Mo	−46	2.67	−11	2.59
tilted C on V	−39	2.44	−44	2.34
tilted C on Mo	−47	2.69	−41	2.58

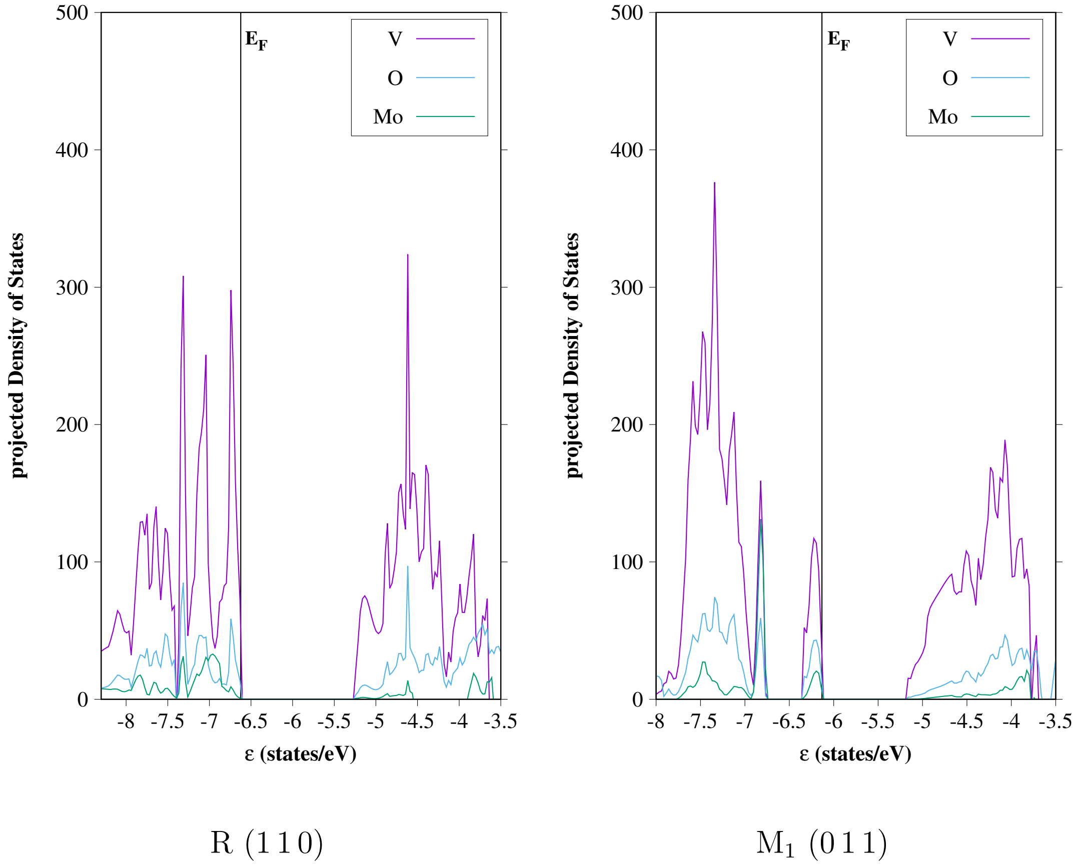

Figure S1: Projected Density of States of alpha frontier orbitals of the R (1 1 0) and M_1 (0 1 1) surfaces without adsorbed CO; V: violet, O: blue, Mo:green.

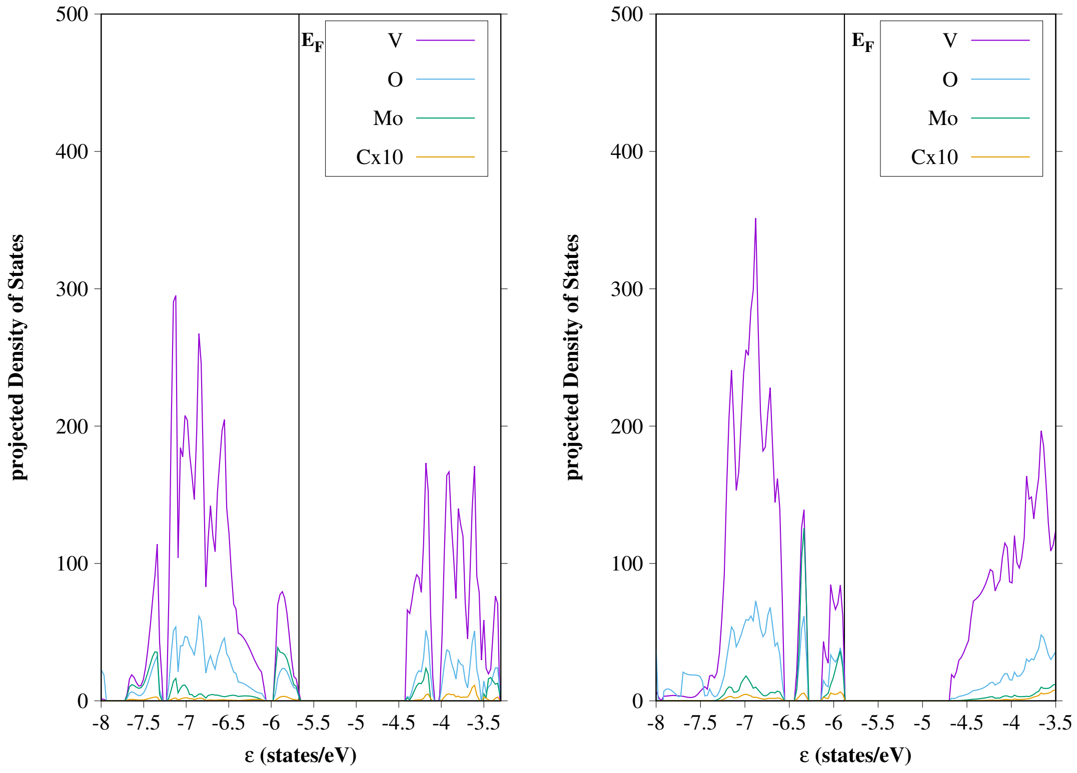

R (1 1 0) M$_1$ (0 1 1)

Figure S2: Projected Density of States of alpha frontier orbitals of the R (1 1 0) and M$_1$ (0 1 1) surfaces with adsorbed CO on the V-Atom; V: violet, O: blue, Mo:green, C: yellow. The intensity of the states of the C-atom have been increased by a factor of 10.

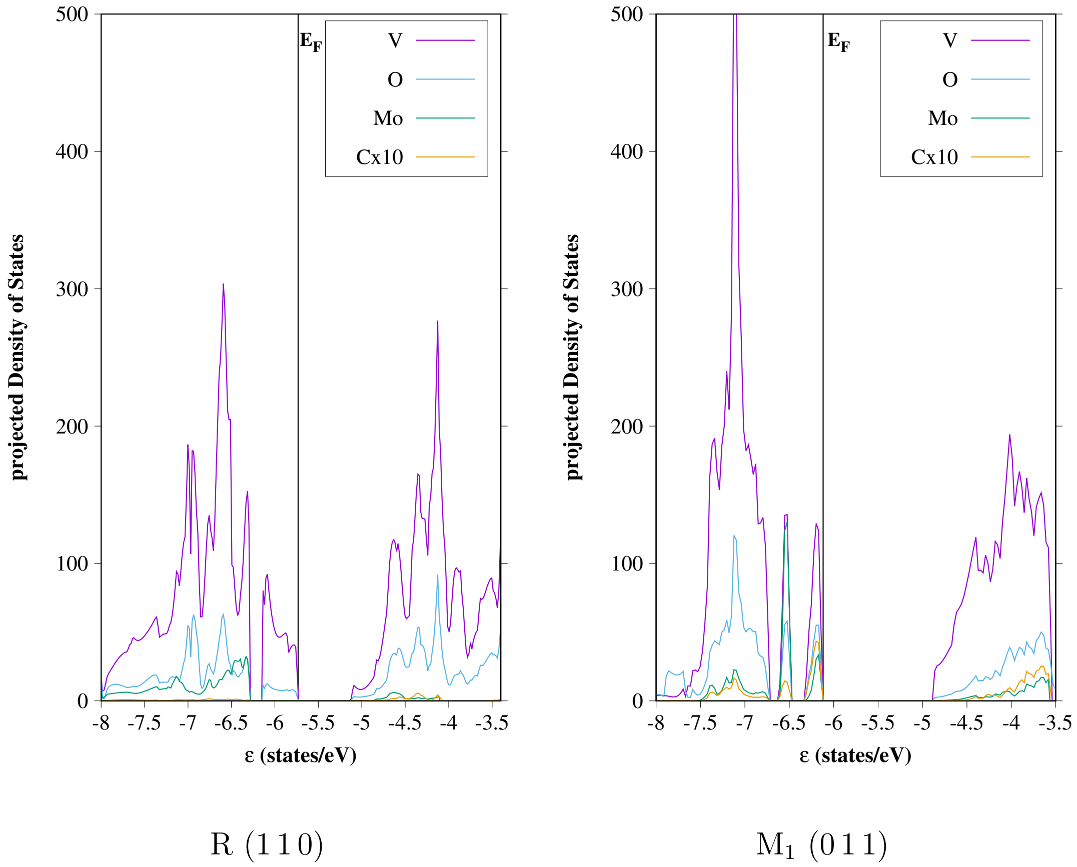

Figure S3: Projected Density of States of alpha frontier orbitals of the R (1 1 0) and M_1 (0 1 1) surfaces with adsorbed CO on the V-Atom with fixed spin(18); V: violet, O: blue, Mo:green, C: yellow. The intensity of the states of the C-atom have been increased by a factor of 10.

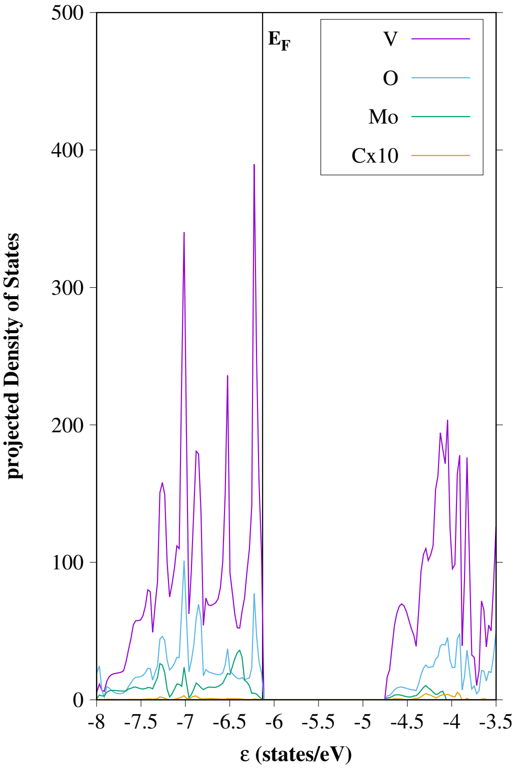

R (1 1 0)

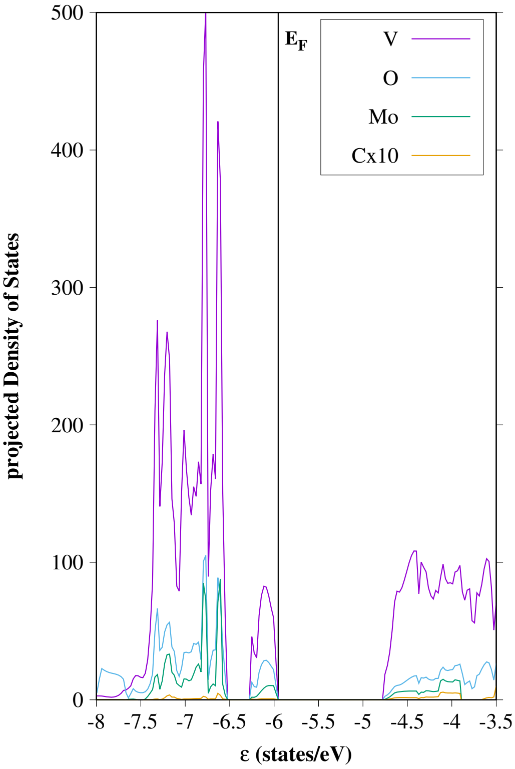

M_1 (0 1 1)

Figure S4: Projected Density of States of alpha frontier orbitals of the R (1 1 0) and M_1 (0 1 1) surfaces with adsorbed CO on Mo-Atom; V: violet, O: blue, Mo:green, C: yellow. The intensity of the states of the C-atom have been increased by a factor of 10.

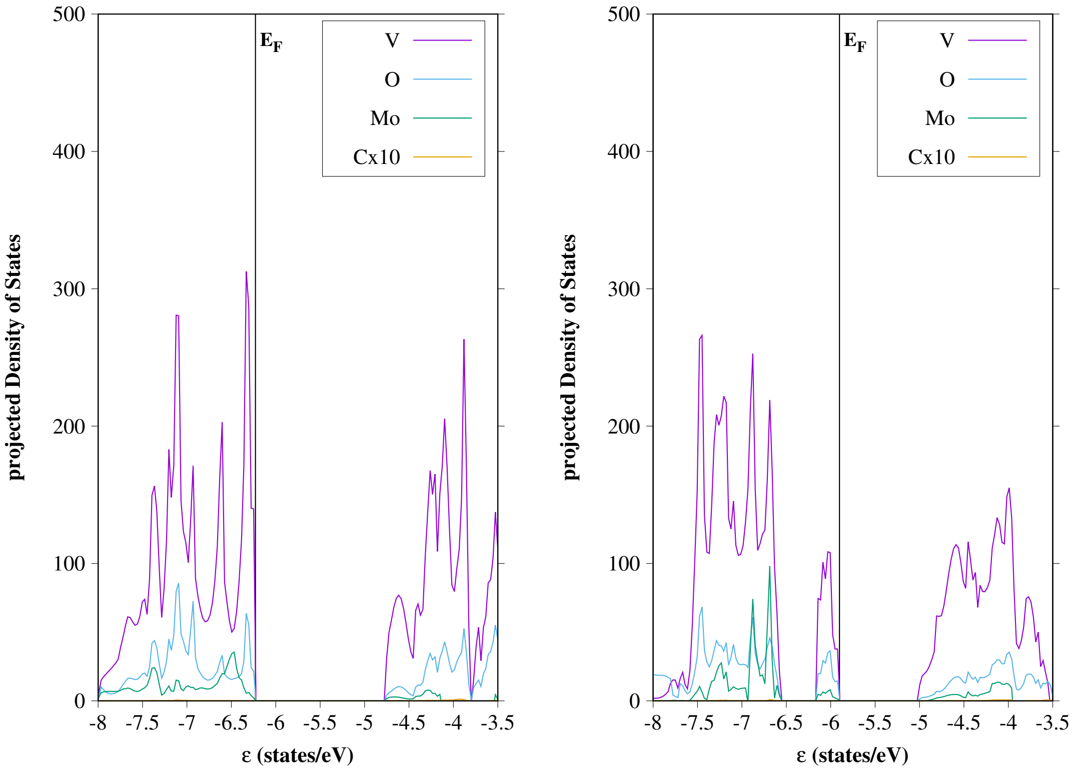

R (1 1 0) M_1 (0 1 1)

Figure S5: Projected Density of States of alpha frontier orbitals of the R (1 1 0) and M_1 (0 1 1) surfaces with the oxygen of CO adsorbed on the Mo-Atom; V: violet, O: blue, Mo:green, C: yellow. The intensity of the states of the C-atom have been increased by a factor of 10.

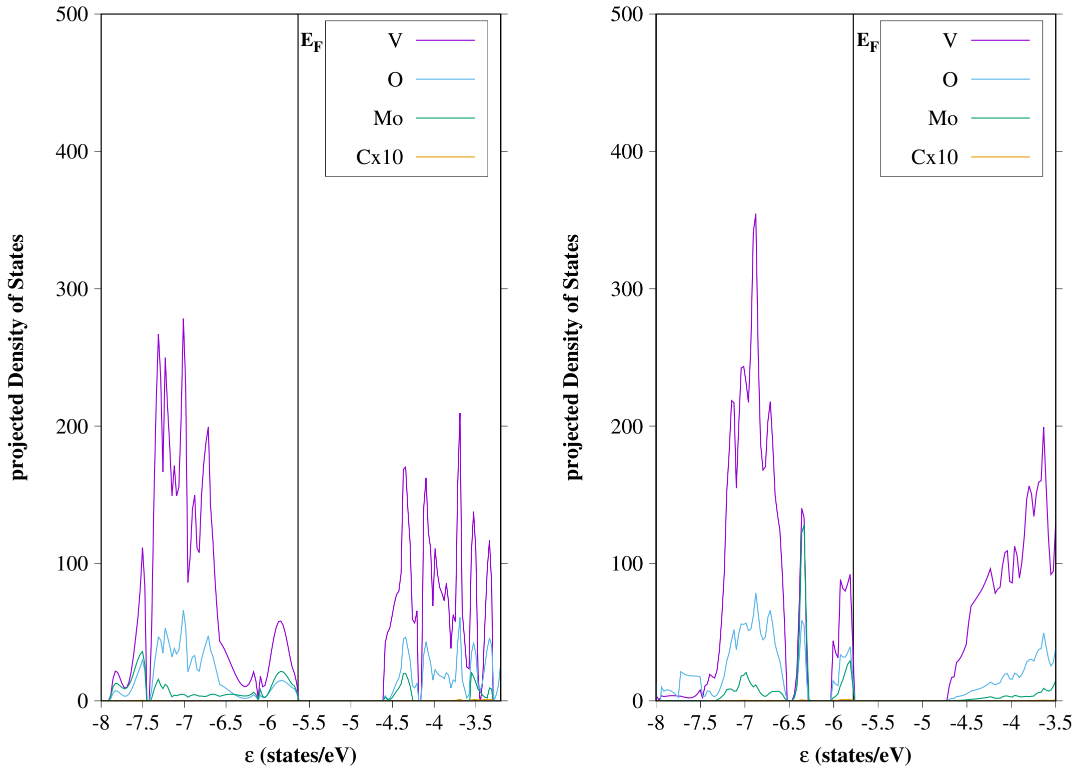

R (1 1 0) M$_1$ (0 1 1)

Figure S6: Projected Density of States of alpha frontier orbitals of the R (1 1 0) and M$_1$ (0 1 1) surfaces with the oxygen of CO adsorbed on the V-Atom; V: violet, O: blue, Mo:green, C: yellow. The intensity of the states of the C-atom have been increased by a factor of 10.

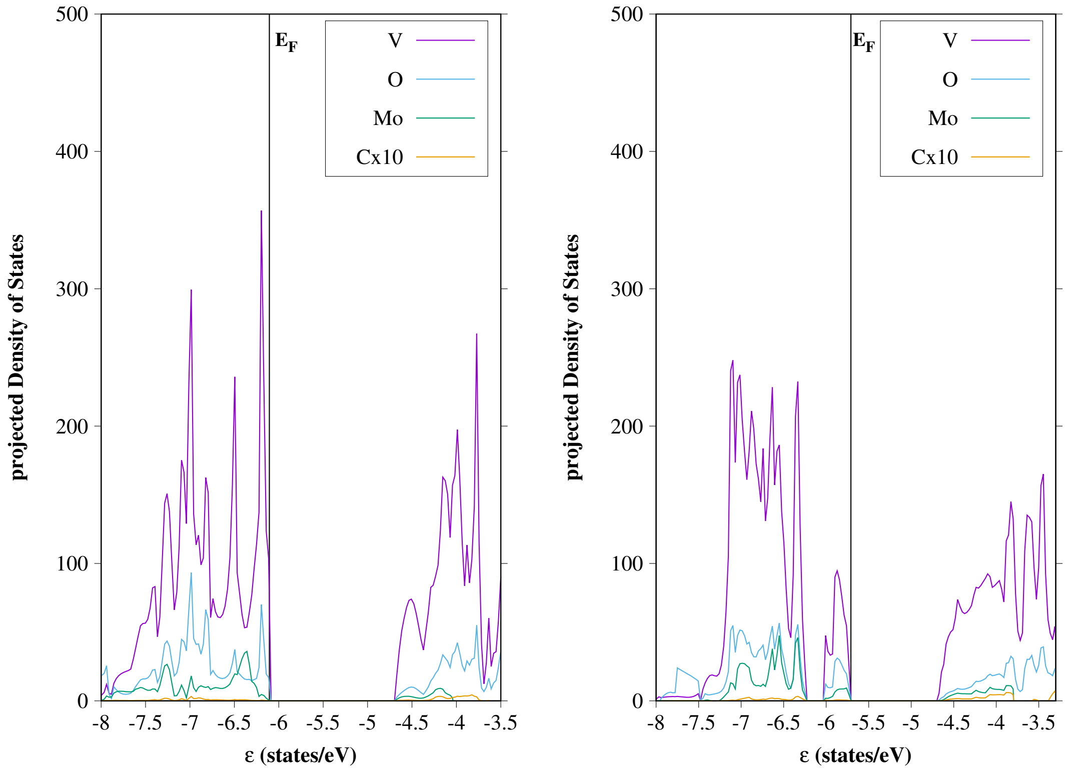

R (1 1 0) M$_1$ (0 1 1)

Figure S7: Projected Density of alpha frontier orbitals of States of the R (1 1 0) and M_1 (0 1 1) surfaces with CO adsorbed rotated on the Mo-Atom; V: violet, O: blue, Mo:green, C: yellow.

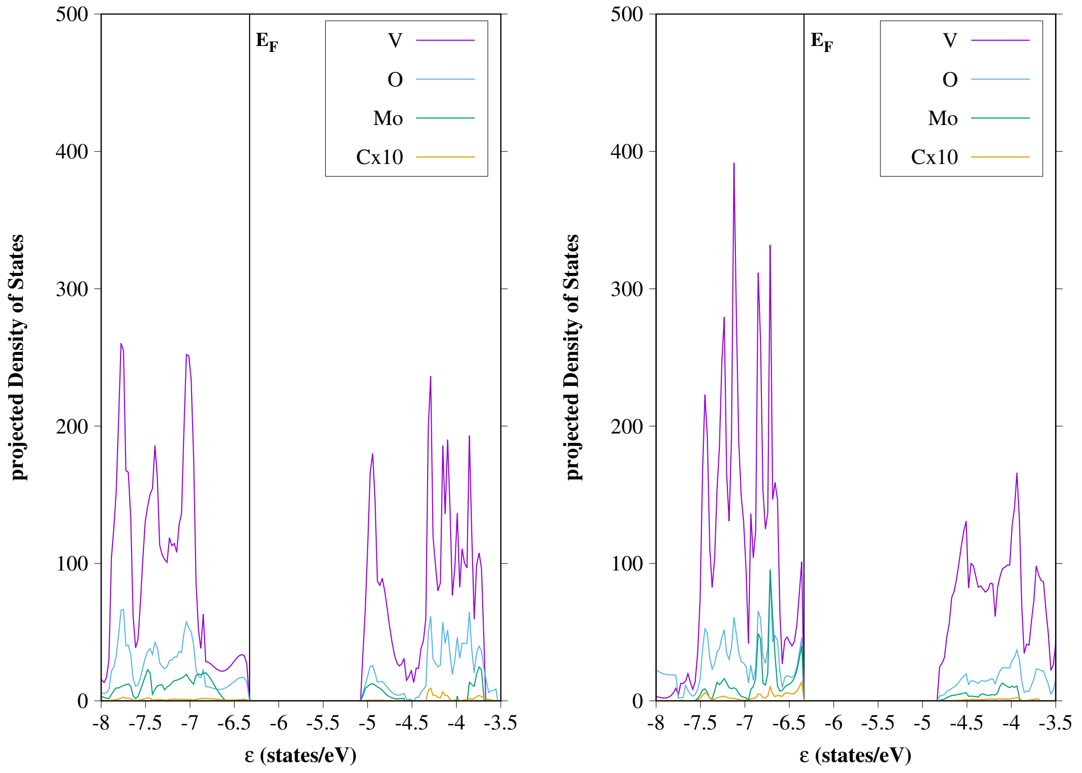

R (1 1 0) M$_1$ (0 1 1)

Figure S8: Projected Density of States of alpha frontier orbitals of the R (1 1 0) and M$_1$ (0 1 1) surfaces with CO adsorbed rotated on the V-Atom; V: violet, O: blue, Mo:green, C: yellow. The intensity of the states of the C-atom have been increased by a factor of 10.

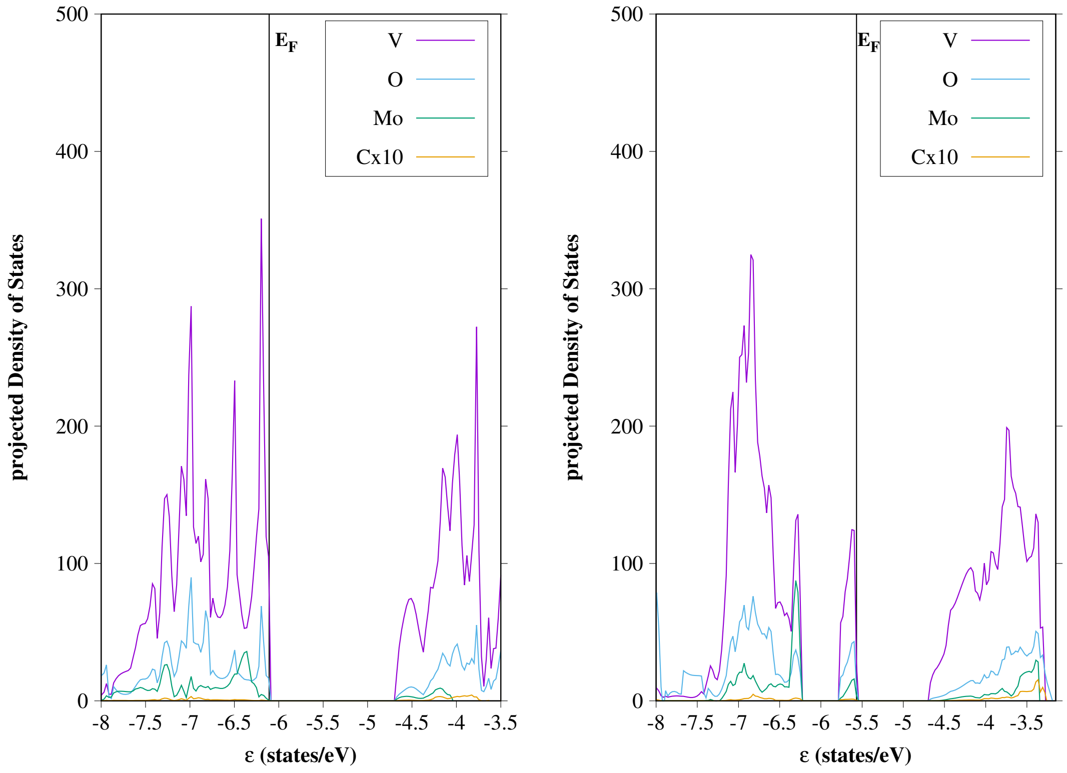

R (1 1 0) M$_1$ (0 1 1)

Figure S9: Projected Density of States of alpha frontier orbitals of the R (1 1 0) and M$_1$ (0 1 1) surfaces with CO side adsorbed on the V-Atom; V: violet, O: blue, Mo:green, C: yellow. The intensity of the states of the C-atom have been increased by a factor of 10.

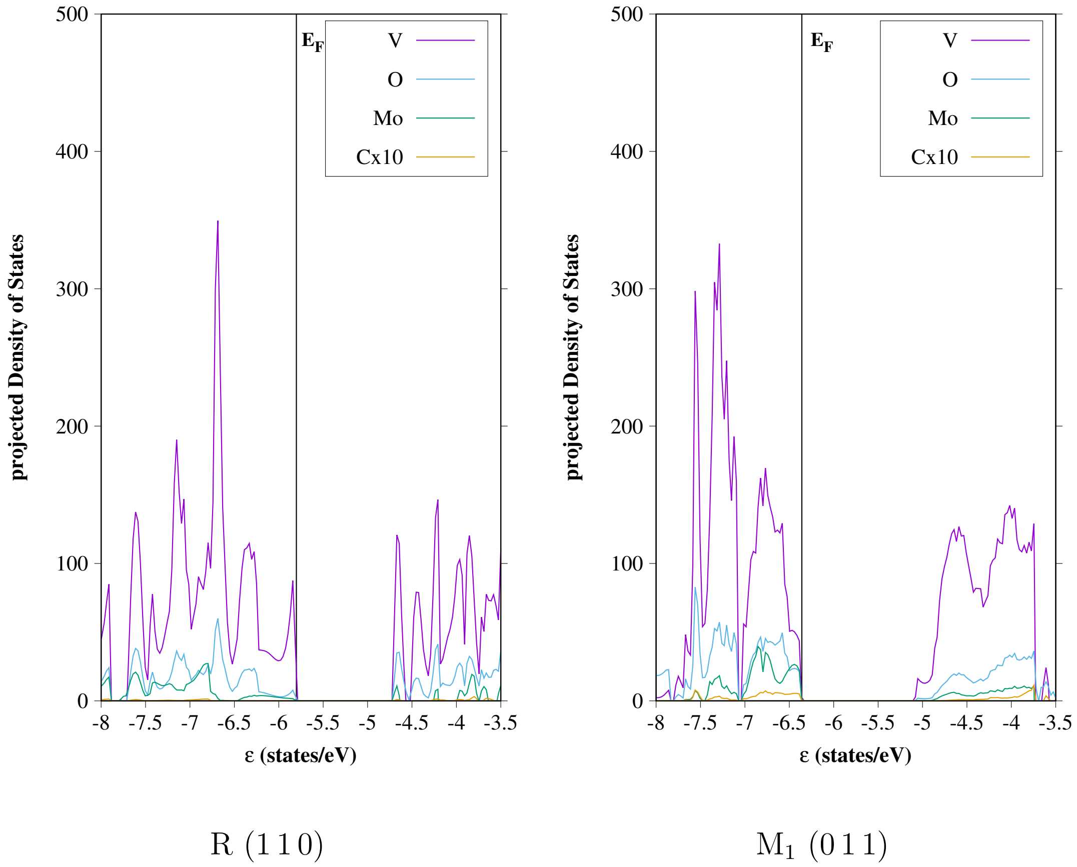

Figure S10: Projected Density of States of alpha frontier orbitals of the R (1 1 0) and M_1 (0 1 1) surfaces with CO side adsorbed on the V-Atom; V: violet, O: blue, Mo:green, C: yellow. The intensity of the states of the C-atom have been increased by a factor of 10.

References

[1] B. Stahl and T. Bredow, Journal of Computational Chemistry **41**, 258 (2020).

[2] T. Koethe, Z. Hu, M. Haverkort, C. Schüßler Langeheine, F. Venturini, N. B. Brookes, O. Tjernberg, W. Reichelt, H. H. Hsieh, H.-J. Lin, et al., Physical Review Letters **97**, 116402 (2006).

[3] F. Pintchovski, W. Glaunsinger, and A.Navrotsky, Journal of Physics and Chemistry of Solids **39**, 941 (1978).

[4] I. Kylänpää, J. Balachandran, P. Ganesh, O. Heinonen, P. R. C. Kent, and J. T. Krogel, Phys. Rev. Materials **1**, 065408 (2017).

[5] J. B. Goodenough, Journal of Solid State Chemistry **3**, 490 (1971).

[6] J. M. Longo and P. Kierkegaard, Acta Chemica Scandinavica **24**, 420 (1970).

[7] A. Akroune, J. Claverie, A. Tazairt, G. Villeneuve, and A. Casalot, Physica Status Solidi (a) **89**, 271 (1985).
